# A Novel HPLC-Based Method to Investigate on RNA after Fixation

**DOI:** 10.3390/ijms21207540

**Published:** 2020-10-13

**Authors:** Paolo Fattorini, Cristina Forzato, Domenico Tierno, Eleonora De Martino, Eros Azzalini, Vincenzo Canzonieri, Giorgio Stanta, Serena Bonin

**Affiliations:** 1 DSM-Department of Medical Sciences, University of Trieste, 34149 Trieste, Italy; fattorin@units.it (P.F.); domenico.tierno@phd.units.it (D.T.); edemartino@units.it (E.D.M.); eazzalini@units.it (E.A.); vcanzonieri@cro.it (V.C.); stanta@impactsnetwork.eu (G.S.); 2 Department of Chemical and Pharmaceutical Sciences, University of Trieste, 34127 Trieste, Italy; cforzato@units.it; 3 Doctorate of Nanotechnology, University of Trieste, 34100 Trieste, Italy; 4 Pathology Unit, IRCCS CRO Aviano-National Cancer Institute, 33081 Aviano, Italy

**Keywords:** RNA, fixation, nuclease digestion, nucleotide-monophosphate, HPLC, degradation, modification, hm-NMP, ISO standards

## Abstract

RNA isolated from fixed and paraffin-embedded tissues is widely used in biomedical research and molecular pathology for diagnosis. In the present study, we have set-up a method based on high performance liquid chromatography (HPLC) to investigate the effects of different fixatives on RNA. By the application of the presented method, which is based on the Nuclease S1 enzymatic digestion of RNA extracts followed by a HPLC analysis, it is possible to quantify the unmodified nucleotide monophosphates (NMPs) in the mixture and recognize their hydroxymethyl derivatives as well as other un-canonical RNA moieties. The results obtained from a set of mouse livers fixed/embedded with different protocols as well from a set of clinical samples aged 0 to 30 years-old show that alcohol-based fixatives do not induce chemical modification of the nucleic acid under ISO standard recommendations and confirm that pre-analytical conditions play a major role in RNA preservation.

## 1. Introduction

Clinical samples from surgery and biopsy procedures are fixed for histopathological analysis. The aim of fixation is to preserve tissues permanently in as lifelike a state as possible and to make it possible to generate thin stained sections. In the past, an optimal morphological preservation was the sole requirement for fixation, whereas nowadays nucleic acids preservation for onco-pathology has also become an important requisite for gene expression profiling and sequencing aimed at defining reliable diagnostic and prognostic parameters [1]. The 4% aqueous solution of formaldehyde (formalin) is the most widely used fixative in histopathology. However, in the past also Bouin’s solution was also applied in certain hospitals for its ability to preserve some morphological details, such as nuclear conformation [2]. In recent years, because of the need of higher quality biomolecules, commercial alcohol-based fixatives have been developed, such as RCL2 [3], FineFix [4], Paxgene [5]. In the above scenario, it is well-known that in formalin-fixed and Bouin’s fixed samples the quality of nucleic acid is lower than in alcohol-based fixatives [4,5,6] and that formalin fixation has led to the formation of mono-methylol adducts (hydroxymethyl derivatives) in nucleic acids and proteins and cross-links between them [7]. Nonetheless, for other fixatives, included Bouin’s solution, their effect in modifying nucleic acids remains unknown and the amount of un-modified nitrogenous bases in the extracts have not been taken into account.

We here describe a novel method based on high performance liquid chromatography (HPLC) that is aimed at investigating on the amount of unmodified nitrogenous bases in RNA extracts obtained from different procedures of fixation.

## 2. Results

**Figure 1:**
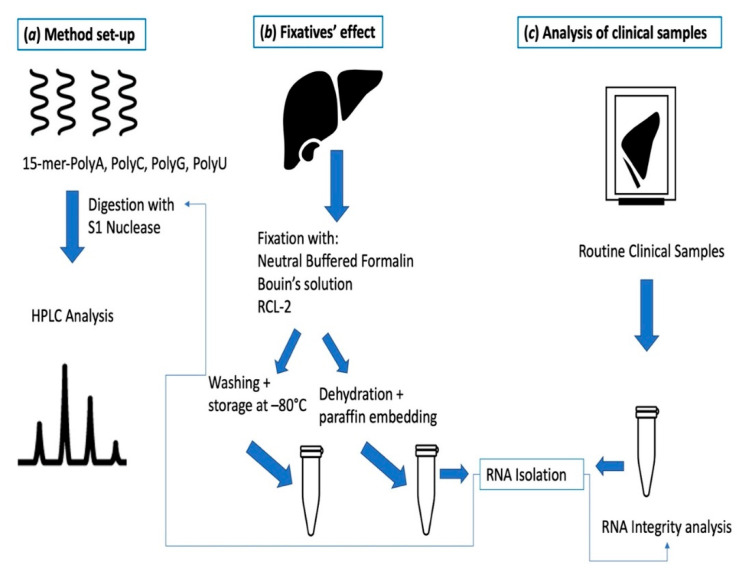
Design of the study: (**a**) Method set-up for the optimization of the digestion conditions and HPLC separation using 15-mer-mono-oligonuclotides; (**b**) Study on fixatives’ effect based on the treatment of the same tissues (mouse livers) with 3 different fixatives and application of the HPLC method to RNA extracts; (**c**) Application of the HPLC method to RNA obtained from routine clinical samples. In sections (**b**,**c**), after isolation, RNA is parallelly submitted to digestion followed by HPLC analysis and to integrity analysis.

To set-up the HPLC-based method to detect unmodified nucleotide monophosphates (NMPs) in RNA extracts, we first set-up the experimental conditions to quantify each unmodified NMP by HPLC and subsequently we investigated methods to degrade RNA into NMPs. The study design is divided into three main sections, as described in Figure 1.

During the procedure’s set-up, hydroxymethyl derivatives of the NMPs were also synthetized as reported in the Appendix A. They were characterized by ^1^H NMR and by HPLC (Appendix A). Their synthesis sought to prove that the hydroxymethyl derivatives (hm-derivatives) of the NMP do not overlap with those that remained unmodified during HPLC analysis.

### 2.1. HPLC Analysis-Quantification of NMPs and Resolution of NMPs and hm-NMPs

A HPLC method was set-up to separate both mononucleotides and their hydroxymethyl derivatives as described in the material and methods section. In order to quantify unmodified NMP, calibration curves with correlation coefficients R^2^ > 0.999 were obtained for each NMP in tests carried out at least in triplicate. The recovery percentage of each NMP and results of the calibration analysis are reported in Table 1.

**Figure 2:**
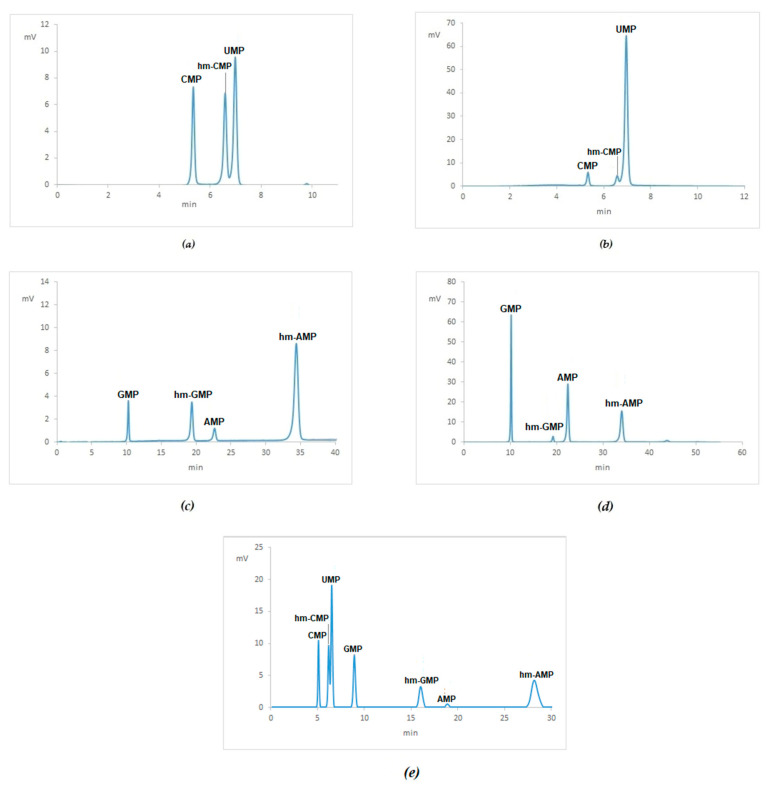
Resolution of NMPs and hm-NMPs by HPLC analysis: (**a**) Mixture of CMP and UMP treated with 4% neutral buffered formaldehyde; (**b**) the same mixture of (**a**) with the addition of UMP; (**c**) mix of GMP and AMP treated with 4% neutral buffered formaldehyde; (**d**) the mixture of (**c**) with the addition of AMP and GMP; (**e**) mixture of the four NMPs treated with 4% neutral buffered formaldehyde.

Formalin fixation modifies nucleic acids by the addition of a mono-methylol group (CH_2_-OH) to the nitrogenous bases [7]. Synthetic hm-derivatives (see Appendix A) were synthetized and submitted to HPLC analysis (see Table 2 and Figure A2). To verify the resolution of NMPs and their hm-derivatives, based on the treatment of NMPs with 4% neutral buffered formalin, a mixture of UMP and CMP/hm-CMP (Figure 2a), and AMP/hmAMP and GMP/hm-GMP (Figure 2c) were submitted to HPLC analysis. Furthermore, spiking the specific NMP to hm-derivatives as shown in Figure 2b for UMP and CMP/hm-CMP and in Figure 2d for AMP/hm-AMP and GMP/hm-GMP, respectively. Overall, the resolution of NMPs and hm-NMPs is shown in Figure 2e where a mixture of the four NMPs and their hm-NMPs was successfully separated by HPLC. Of note, UMP does not apparently react with 4% neutral buffered formalin, as shown in the 1H-NMR spectra in the Appendix A and in chromatograms in Figure 2a,b,e.

The retention times of the four NMP and the three hm-NMP are reported in Table 2, which shows that the CV % was always <7.3% in intra-session experiments, whereas higher values in inter-session were due both to the exhaustion of the solid phase and to the effect of room temperature in a non-thermostated HPLC apparatus.

### 2.2. RNA Digestion Set-Up

**Figure 3:**
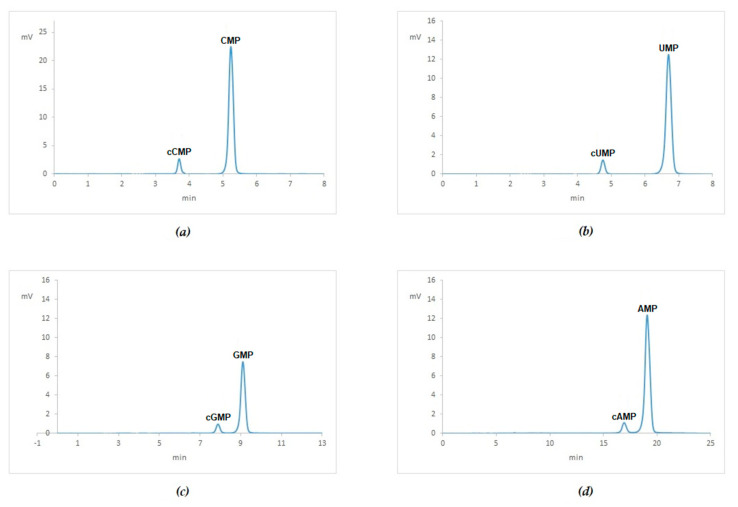
HPLC analysis of digested 15-mer mono-oligonucleotides: Chromatogram of digestions of polyC (**a**), polyU (**b**), polyG (**c**), polyA (**d**).

Hydrolysis of the nucleic acids can be achieved both by physical-chemical and enzymatic procedures [8,9,10,11,12]. In keeping with the aim of the present study, physical-chemical procedures involving samples heating were avoided as they can release mono-methylol adducts from the RNA chain thus interfering with the reliability of the HPLC analysis. Consequently, enzymatic reactions with S1 Nuclease (Promega) which cleaves the RNA molecule to 5′-mono-nucleotide were preferred. Digestion conditions were assessed using 15-mer mono-oligonucleotides, namely polyA, polyC, polyG and polyU followed by HPLC separation as shown in Figure 3 and Table 3.

Figure 3 shows that after incubation with S1 nuclease of the 15-mer mono-oligonucleotides they were digested into the specific NMP. Minimal amount of the cyclic forms of each NMP were also detected [13].

To confirm our findings, a standard RNA, as provided by Celbio, after digestion with S1 nuclease returned the expected four peaks at the defined retention times. For the following quantification analyses, a reaction blank including a solution of S1 Nuclease (Promega) in 1× digestion buffer was injected into HPLC to identify peaks related to non-template control as shown in Appendix A—Figure A3. Those signals were subtracted from the following chromatograms for quantification.

### 2.3. Fixatives Conditions in Mouse Livers

The aim of the present study was to apply the HPLC method to fixed samples. To this end, mouse livers were used for the analysis set-up. Mouse livers were submitted to different fixation procedures for 24 h, namely neutral buffered formalin, Bouin’s solution and RCL2^®^, with and without paraffin embedding in comparison to untreated samples (frozen specimen) following ISO standard for pre-analytical processes [14].

**Figure 4:**
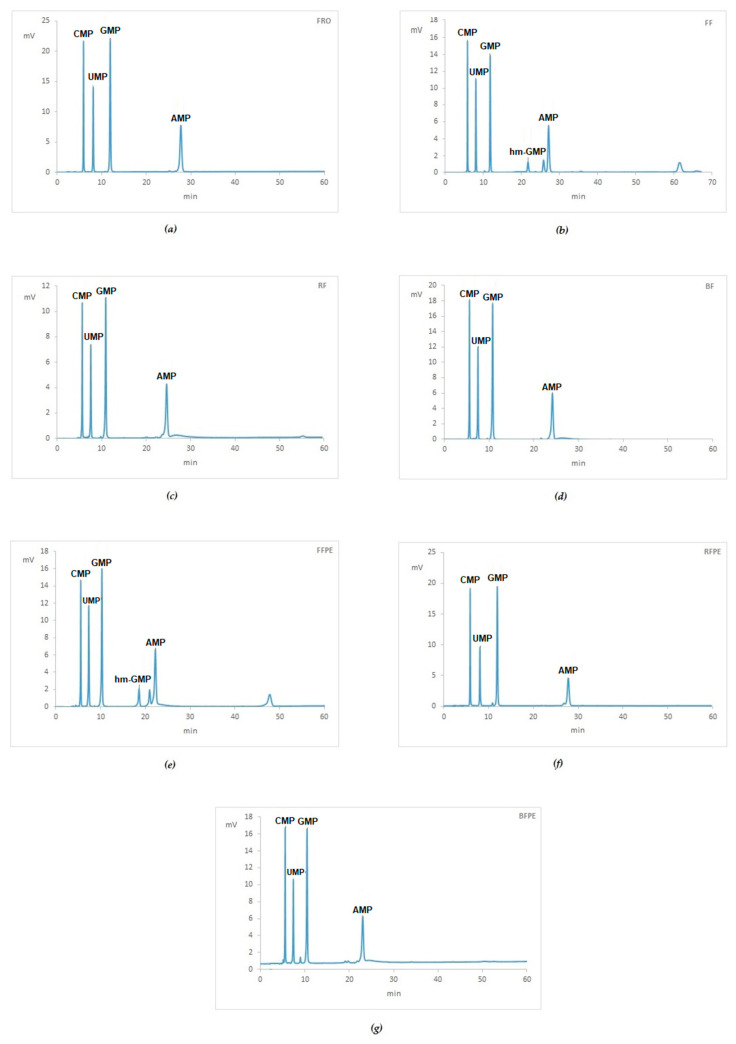
HPLC analysis of mouse livers submitted to different treatments: Chromatograms of (**a**) Fresh frozen (Fro); (**b**) 24 h in neutral buffered formalin (FF); (**c**) 24 h in RCL2 (RF); (**d**) 24 h in Bouin’s solution (BF); (**e**) 24 h in neutral buffered formalin and paraffin-embedded (FFPE); (**f**) 24 h in RCL2 and paraffin-embedded (RFPE); (**g**) 24 h in Bouin’s solution and paraffin-embedded (BFPE). Uncanonical RNA moieties are detected in mouse livers treated with 4% neutral buffered formalin.

Each HPLC analysis was carried out in triplicate loading 600–700 ng of digested RNA in each injection. Chromatograms, where peaks related to the blank were subtracted, are shown in Figure 4. In mouse livers treated with 4%, neutral buffered formalin different digestion patterns were detected showing more than four canonical peaks of unmodified NMPs (Figure 4b,e). In Table 4 are reported the percentage area of unmodified nucleotide monophosphates detected in the chromatograms.

Regarding hm-NMPs, the chromatograms in Figure 4 reveal among peaks, only hm-GMP in samples treated with formalin (Figure 4b,e, respectively). The ratio of the peaks areas between hm-GMP and GMP was about 0.21. No other peaks referring to hm-nucleotide monophosphates were recorded in FF and FFPE samples. In RCL2 treated samples as well as in Bouin’s treated samples, peaks attributable to hm-derivatives were not identified.

### 2.4. RNA Quantification and Integrity from Mouse Livers

RNA purity was measured through the A260/280 and A260/230 ratios by the NanoDrop™ ND-1000. The mean A260/280 ratio was 1.98 (range 1.70–2.10), reaching the lower value in BF liver specimen. A260/230 ratio was similar in all samples with an average of 2.11 (range 2.07–2.14) as reported in Table 5.

Integrity of RNA extracts was investigated by the Agilent 2100 Bioanalyzer (Agilent Technologies; Santa Clara, CA, USA) recording the RIN number and quantifying the different RNA fragments (see Supplementary Materials for the Gel-like images of the BioAnalayzer runs). Mean RIN number was 2.1 for fixed as well as fixed and embedded samples, but only the Bouin’s fixed sample did not return any value and RCL2 fixed and embedded sample had the lowest RIN value (Table 5). As already shown [15] electrophoretic approaches are not particularly sensitive to more seriously degraded RNA such as the ones from fixed samples, although PERM [16] and DV [17] algorithms have been proposed for a better application of the method. RIN values reflect the quality of rRNA rather than mRNA.

**Figure 5:**
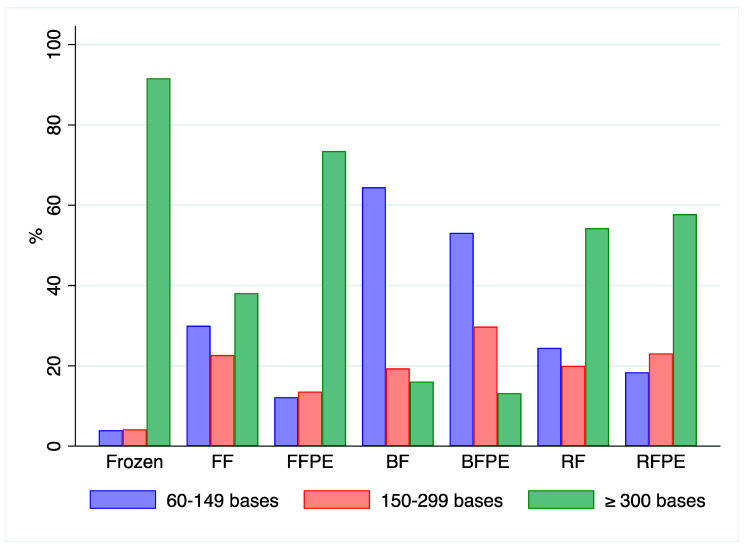
Analysis of RNA fragments length: Bar plots representing the distribution of 60–149, 150–299 bases and equal to or over 300 bases fragments by BioAnalyzer method in fresh frozen, FF formalin-fixed, FFPE formalin-fixed and paraffin-embedded, BF Bouin’s solution fixed, BFPE Bouin fixed and paraffin-embedded, RF RCL2 fixed and RFPE RCL2 fixed and paraffin-embedded mouse livers. A higher fragmentation is observed in Bouin’s solution fixed samples (BF and BFPE, respectively).

The relative amount of RNA stretches was retrieved by the fragment size analysis using the Agilent 2100 Bioanalyzer. Stretches of 60–149 nt prevailed in Bouin’s fixed mouse livers, in spite of the embedding process. On the contrary, for formalin and RCL2 a comparable amount of 60–149 nt and 150–299 nt fragments were observed, as shown in Figure 5. In frozen sample, most fragments (85%) were over 300 bases long.

**Figure 6:**
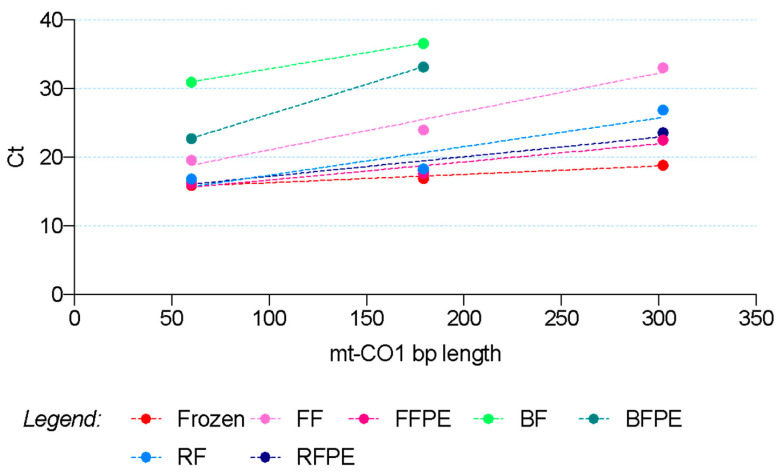
RT-qPCR of mouse livers samples: Linear regression lines obtained plotting the threshold cycle (Ct) by RT-qPCR for mitochondrially encoded cytochrome c oxidase I (mt-CO1) versus the amplicon length (bases) in mouse livers extracts. In Bouin’s solution, fixed samples (BF and BFPE, respectively) fragments of 300 bases were not amplifiable.

In addition, the RNA integrity was investigated in mouse livers tissue by amplifying different stretches of the cytochrome C oxidase I, mitochondrial (mt-CO1) gene (NC_005089.1) [18], characterized by different lengths. The linear regression lines were obtained by plotting the Ct versus the amplicon size as shown in Figure 6. RNA stretches of mt-CO1 were amplifiable in all fixed and fixed-paraffin-embedded samples, but Bouin’s ones where the maximum amplifiable size was 179 bases. Furthermore, Bouin’s fixed samples had higher Ct values.

In an overall comparison, only the intercepts resulted as being significantly different among pre-analytical treatments (*p* < 0.0001, Table 6). This notwithstanding, in performing a Bonferroni multiple comparison analysis, the BFPE slope was significantly different from the FFPE, RFPE and Frozen ones (*p =* 0.03, *p* = 0.04 and *p* = 0.01, respectively). The FFPE and RFPE slopes were closer to the slope of the fresh frozen sample (Table 6). Overall, the performance of RT-PCR resulted better in fixed and paraffin-embedded specimens in comparison to fixed ones.

### 2.5. Clinical Samples

**Figure 7:**
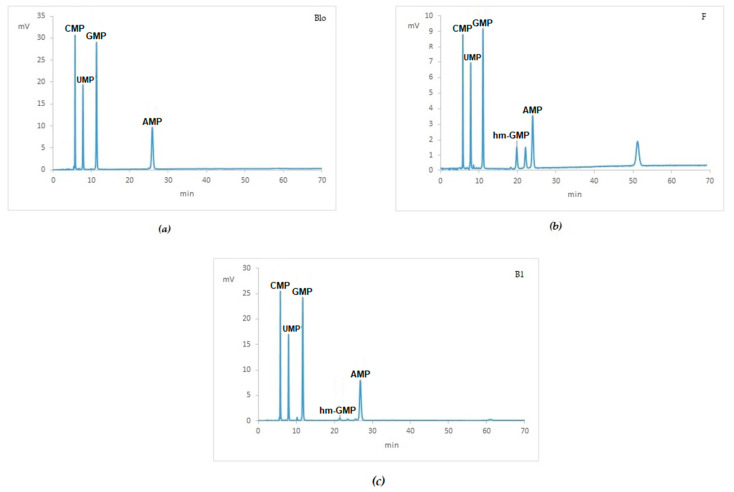
HPLC analysis of routine clinical samples: Chromatograms of (**a**) RNA from peripheral blood; (**b**) FFPE (ovarian cancer); (**c**) BFPE (ovarian cancer). Fixed and paraffin-embedded ovarian cancers display more complex patterns of digestion.

The HPLC method was finally applied to routine clinical samples, including two BFPE high-grade serous ovarian cancer samples (B1 and B2), 10 FFPE tissues of different cancer types and three RNAs obtained from peripheral blood. Figure 7 shows examples of HPLC separation and the percentage of each of the canonical NMP is reported in Table 7.

All the samples showed a reduced amount of the canonical NMP, down to 31% in a 28-years old cervical uterine cancer. However, no relationship was found between the aging of the samples and the percentage of the canonical NMPs.

After the de-modification step (which was carried out at 80 °C for one hour in all fixed samples), only hm-GMP was detected in the chromatogram with different extent in all FFPE samples as well in BFPE samples. The ratio among peak areas (hm-GMP vs. GMP) was 0.02 in Bouin’s sample 1, 0.03 in Bouin’s sample 2, an up 0.28 in FFPE tissues. Furthermore, peaks at higher retention times (≥40 min) were recorded in all the samples, with major amplitude in FFPE samples likely related to dimers (methylene di-adducts) between nitrogenous bases as resulting from the cross-linking process.

### 2.6. RNA Quantification and Integrity of Clinical Samples

The integrity of RNA extracts was investigated by the Agilent 2100 Bioanalyzer (Agilent Technologies; Santa Clara, CA, USA) recording the RIN number and quantifying the different RNA fragments. Gel-like images of the BioAnalayzer runs are available in the Supplementary Materials. Mean RIN number of fixed samples was 2.2 and the colon cancer 1 sample had non-assessable RIN value (Table 7).

**Figure 8:**
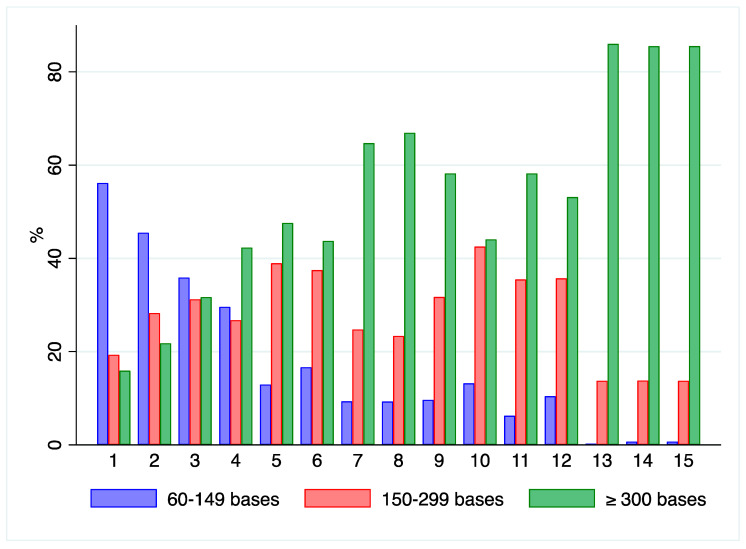
Analysis of RNA fragments length: Bar plots representing the distribution of 60–149 and 150–299 bases and equal to or over 300 bases fragments by BioAnalyzer method in clinical samples. Cases follow the order of Table 7: samples 1,2—BFPE samples; 3–12 FFPE samples; 13–15—Blood samples. BFPE samples show higher RNA degradation.

In all fixed samples, the most representative fractions were related to fragments of 60–299 nucleotides as reported in Figure 8. In Bouin’s fixed and embedded samples (1–2) the 60–149 nucleotides fragments were more represented than the 150–299 nucleotides fractions, while on average in formalin-fixed samples (3–12) the 150–299 nucleotides fraction prevailed. RNA from blood specimens had longer fragments as shown in Figure 8 (samples 13–15) and confirmed by the associated RIN numbers.

## 3. Discussion

Tissue fixation is a compulsory step for preserving cellular and tissue morphology for pathological examination. Fixation by itself introduces artifacts, involving some chemical modification of tissue components used to prevent their loss during tissue processing [19]. With respect to RNA preservation, it has already been reported that both formalin and alcohol-based fixatives have damaging effects on RNA preservation, and that the entity of those effects is fixative-specific [4]. In the present study, we propose a HPLC-based method as a tool to investigate on the RNA composition after fixation by analyzing the amount of canonical (e.g., unmodified) nucleotide monophosphates (NMP).

The method *per se* does not reveal the quality of RNA molecules in terms of fragment lengths, but rather it allows investigating the chemical integrity of each of the four canonical NMP of the RNA. The method in fact allows both identification and quantification of the NMPs released by the enzymatic digestion with nuclease S1 from as low as 600–700 ng of RNA. Consequently, in agreement with our expectation, only the four canonical NMPs were detected in our control samples. In addition, although non-quantifiable owing to the unavailability of commercial standards, home-synthesized hydroxymethyl NMPs (e.g., the most abundant classes of formaldehyde-deriving products) were efficiently resolved as well.

Out of the hm-NMPs, only hm-GMP was detected in our series of FFPE mouse liver samples as well as in clinical samples. Among hydroxymethyl nucleotide monophosphates, hm-AMP was reported to account around 40% of Adenines [7], but it was not identified at the expected retention time in our chromatograms. The fact that hm-NMPs have not been detected in our HPLC analyses is not surprising because our RNA isolation method has included a de-modification step at 80 °C. Several authors, have indeed claimed that heat-mediated de-modification of RNA from FFPE tissues mitigates the adverse effects of formalin on RNA [7,20,21], reporting that the de-modification step allows the removal of about 72.5% of modification, presumably methylol addition [7]. Furthermore, hydroxymethyl derivatives of dNMPs have been reported to be quite labile and unstable, therefore also for NMPs derivatives it is possible that their absence in the chromatograms could be related to their instability [22,23]. This hypothesis is supported also by our chromatograms without the de-modification step (Appendix C, Figure A4), where the hm-AMP and hm-CMP were not detectable. Regarding the rate of modification of NMPs, data in the literature are heterogeneous with the limitation that most studies analyzed DNA and reported data from in vitro reaction and not from ex vivo analysis. Masuda and co-workers found that Adenine and Cytosine were the most modified nitrogenous bases after in vitro treatment of RNA with formaldehyde [7], while Karmakar and colleagues focused their study mostly on AMP and dAMP [24]. Our results on the in vitro conversion of NMPs by formaldehyde rank AMP modification (66%) above CMP (50%) and GMP (36%). In agreement with Masuda et al. UMP has not been modified by formaldehyde addition [7]. Our ranking of hm-AMP above hm-CMP and hm-GMP agrees with Beland who reported the same ranking for the hydroxymethyl-dNMPs. For DNA adducts, the order mirrors also their ease of formation and their relative stability [22]. The fact that we detected only hm-GMP is surprising and can find a possible explanation in the more complex reaction of formaldehyde with the derivatives of Guanine, with three potential sites of reaction (the two protons of the amino group at C-2, and the proton of the N 1 nitrogen of the heterocyclic ring) [25]. Furthermore, our results showed that the de-modification step can reduce the hm-GMP peak of up to 10 times and it doesn’t influence the RIN value by Agilent Bioanalyzer (see Appendix C, Figure A4 and Figure A5).

In our series of mouse livers, differences were detected in the HPLC profile of formalin-fixed samples when compared to FFPE ones. Higher values of canonical NMPs were assessed in the former (85% vs. 80%) and no peak referring to hm-GMP was detected as well. The latter result could have different explanations. From one side it is possible that the absence of the dehydration process in fixed sample and the subsequent treatment of the sample in aqueous phase for the RNA isolation allows the re-conversion of hm-GMP to GMP, as observed for hm-dAMP [25]. It is also possible that the dehydration process in alcohol may play a role in the conversion of formaldehyde-modified nucleic acids [26], which of course is lacking in pure formalin-fixed samples. Lastly, signals at higher retention times (>40 min) in our chromatograms are likely due to bridging irreversible dimers of amino bases [24] between NMPs or between NMPs and protein residues. A confirmation of that interpretation is given by the presence of that peak only in FFPE liver. Methylene di-adducts are known to be more stable than the hm-NMPs explaining their detectability in both formalin-fixed and FFPE samples [22].

Both formalin and Bouin’s solution are formaldehyde-based fixative, but different patterns of modification were found by HPLC analysis in mouse livers. The amount of unmodified NMP was always higher in Bouin’s fixed samples when compared to formalin-fixed ones (96–100% vs. 80–85%) Bouin’s fixative is made up of picric and acetic acids as coagulant components of the fixative and formaldehyde as cross-linking one [27]. RNA from BS fixed samples resulted more degraded in terms of fragment detection and RT-PCR analyses, indicating a possible ab initio fragmentation due to the low pH of the fixation solution [2,6,27]. The modification of nitrogenous bases in BS fixation is likely avoided or reduced by the acidic pH of the solution [28].

In our series of mouse livers, the quality of samples in terms of RT-PCR seems to be higher in fixed and paraffin-embedded specimens if compared to fixed ones, independently of the fixative used. That apparent discrepancy can find a possible explanation in fixed sample treatment, which were submitted to fixation, washed and frozen at −80 °C. For RNA isolation, they were thawed and submitted to mechanical homogenization. The fact that fixed samples were not dehydrated, maintaining residual water in tissues and that a freezing/thawing cycle was applied together with the heating due to mechanical homogenization might account for that difference. This is further supported by the intrinsic properties of mt-CO1 gene (also known as ERR marker), which has been reported to be resistant to RNAse degradation, but heat-sensitive [15]. That marker had a higher ∆amp in fixed samples in comparison to their correspondent fixed and paraffin-embedded samples (Appendix B, Table A1). Given the same pre-analytical steps before fixation, our analyses on mouse tissues confirm that Bouin’s fixative induces higher fragmentation of RNA, as shown by the absence of amplification of 302 bases stretches as well as by higher level of 60–149 fragments at the BioAnalyzer (Figure 5). Similarly, RCL2^®^ proved to preserve better RNA after fixation assuring about 99% of unmodified NMPs, while this value lowered to 88% after embedding procedure, likely due to the procedure itself [26]. By comparing regression lines for the different pre-analytical procedures in mouse livers, the slopes of the lines were almost similar among treatments, differing only for BFPE livers. This indicates that the efficiencies of PCR systems did not vary among treatments; ergo no particular inhibitory effects were recorded for most of them, but BFPE. The different values of intercepts are indicative of the different starting conditions, namely the sample degradation level due to the fixatives, among pre-analytical processes.

The HPLC method was also applied to a set of aged clinical samples (see Table 7), which showed some differences when compared to mouse livers. Although to a minor extent, hm-GMP signal and peaks at retention times ≥40 min were found also in Bouin’s fixed paraffin-embedded ovarian cancer samples (Figure 7c). This might be due to the pre-analytical conditions at the time of tissue collection accounting for minor composition differences of BS that could have allowed the cross-linking activity of formaldehyde in clinical Bouin’s samples. Furthermore, for mouse livers ISO pre-analytical standard was applied for tissue collection and processing [14] in order to guarantee an optimal quality level in the samples. Nevertheless, tissue embedding was also reported to induce transcriptional artifacts [21,26]. Another concurrent phenomenon is related to tissue storage period in the archive, which is known to influence the quality of nucleic acids from fixed tissues [29,30,31,32,33]. Similarly, differences in terms of hm-GMP amount observed between routine clinical FFPE samples can be ascribed mainly to the diverse pre-analytical procedures.

In conclusion, the HPLC method here described makes it possible to detect the four canonical NMPs of the RNA as well as their hydroxymethyl derivatives. Most importantly, when applied to less than 1 µg of RNA from fixed tissues, it provides valuable data on chemical modification of RNA molecule as produced by fixation processes. It has been possible to demonstrate, indeed, that RCL2^®^, as possibly most alcohol-based fixatives, does not induce chemical modification of the nucleotide monophosphates. In addition, our data support that Bouin’s solution prevents, in ISO standard pre-fixation conditions, the cross-linking activity of formaldehyde. Furthermore, we have shown that hm-GMP is the most represented hm-NMP. We acknowledge as a limitation of this method that it allows the quantification on unmodified NMPs as indirect proof on the non-modification of RNAs, without any additional information on the degradation of the transcripts, which is mandatory for further molecular analysis. Nevertheless, the presented method could easily be applied for analyzing the effect of novel fixatives and/or RNA de-modification procedures. Consequently, these data on the molecular composition of the template, coupled to conventional and newly proposed methods, such as the PERM and DV algorithm from BioAnalyzer for assessing the molecular weight of the templates, are helpful in the evaluation of RT-qPCR- based results that can be achieved from archived fixed and paraffin-embedded samples. Even in long-term stored FFPE tissues, only hm-GMP and di-adducts were found after the de-modification step of the RNA, without correlation with the age of the blocks. The present results stress that pre-analytical procedures play a major role in the quality of the clinical specimens, thus confirming that the use of the ISO standard document [14] guarantees higher quality level samples for molecular investigations.

## 4. Materials and Methods

### 4.1. RNA Digestion

Hydrolysis of the nucleic acids was achieved by enzymatic reactions which cleave the RNA molecule to 5′-mono-nucleotide. Three different enzymes were tested: S1 Nuclease (Promega, Madison, WI 53711-5399, USA; Cat. No. E567B), S1 Nuclease (Invitrogen- Thermo Fischer Scientific, Waltham, MA 02451, USA; Cat. No. 180001-016) and RNAase ONE^TM^ Ribunclease (Promega, Madison, WI 53711-5399, USA; Cat. No. M426A). For assessing the digestion conditions, 5 µg of each 15 bases oligo (polyA, polyC, polyG and polyU, purchased by IDT—Integrated DNA Technologies; Leuven, Belgium) as well 5 µg of a control RNA purchased by Celbio were incubated in 1× working buffer with 50–160 units of enzyme at 37 °C for two hours in a final volume of 50 µL. After digestion, the samples were immediately stored at −20 °C up to HPLC analysis. The following digestion reactions of mouse liver as well as clinical samples were carried out using about 2 µg of RNA with 80 units of S1 Nuclease (Promega, Madison, WI 53711-5399, USA) in the abovementioned conditions.

### 4.2. Fixation Procedure

Four wild type Black Swiss mouse livers were collected at the ICGEB animal house after sacrifice. Each liver was washed in PBS, blotted onto a paper and immediately chilled on wet ice for transport. At the pathology department, livers were subdivided into fragments and submitted to fixation. Each liver tissue was equally represented in the different treatments. According to ISO standard [14] a duplicate of samples for each treatment were fixed for 24 h in neutral buffered formalin, Bouin’s solution and RCL-2 at room temperature in the dark. Afterwards, fixed samples were washed in PBS and a set of samples for each treatment was blotted onto paper and stored at −80 °C up to RNA isolation. The second set of samples was dehydrated and paraffin-embedded following standard procedures [14]. A replicate of fixed samples was obtained during this procedure. Thus, in total, 12 samples (fixed + fixed and paraffin-embedded) were processed for this study.

### 4.3. HPLC Analysis

**Figure A1:**
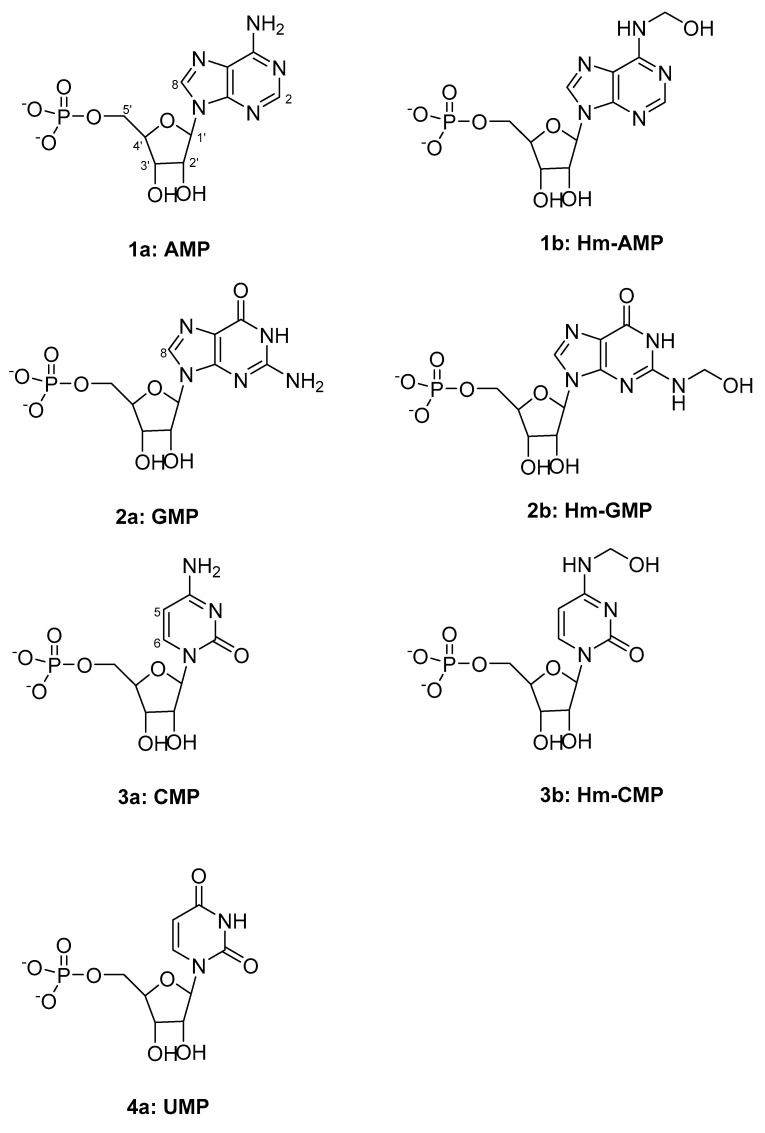
Structure of mononucleotides **1a**–**4a** and hydroxymethyl adducts **1b**–**3b**.

**Figure A2:**
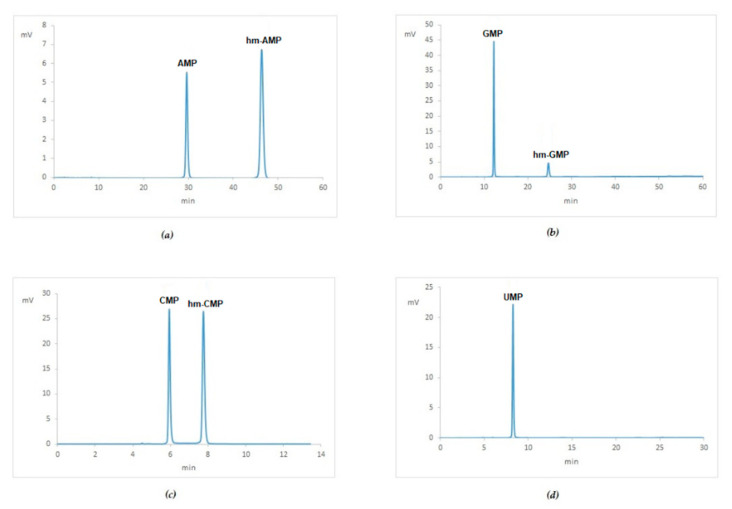
Chromatogram of: (**a**) hydroxymethyl-AMP **1b** (peak 2) and unmodified AMP **1a** (peak 1); (**b**) hydroxymethyl-GMP **2b** (peak 2) and unmodified GMP **2a** (peak 1); (**c**) hydroxymethyl-CMP **3b** (peak 2) and unmodified CMP **3a** (peak 1); (**d**) unmodified UMP **4a**.

**Figure A4:**
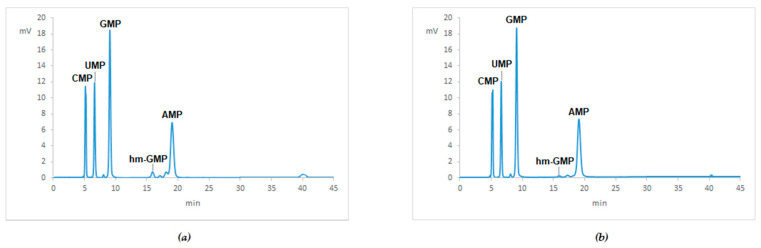
Chromatogram of: (**a**) prostate cancer FFPE tissue without RNA de-modification step and (**b**) with de-modification step.

**Figure A5:**
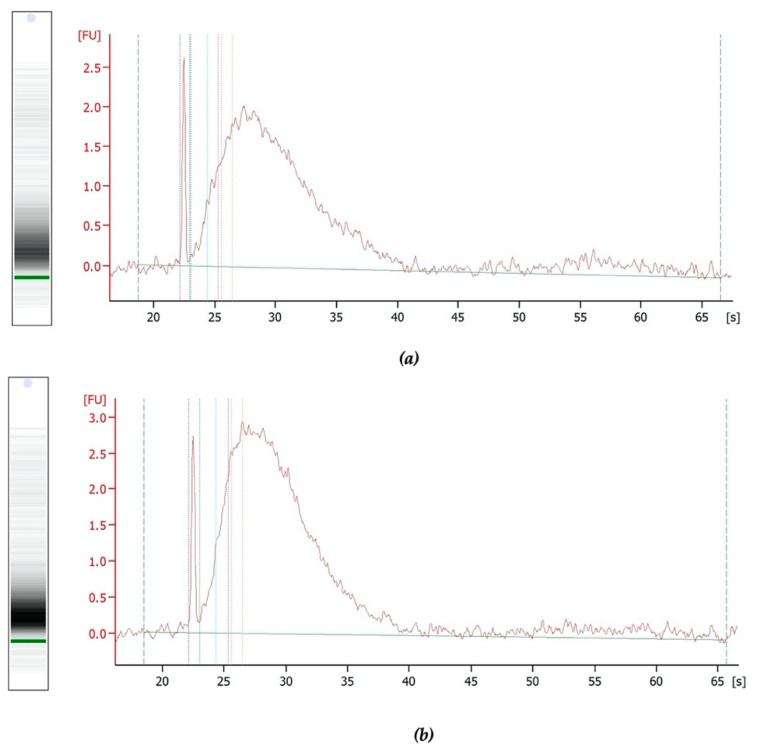
Output of the BioAnalyzer analysis of FFPE prostate cancer tissue (**a**) without RNA de-modification step and (**b**) with de-modification step. The image includes the gel-like image (**left side**) and the electropherogram (**right side**).

HPLC analyses were run on a non-thermostated Agilent series 1100 liquid chromatograph equipped with a Kinetex C18 250 × 4.6 mm 5 µm 100 Å (Phenomenex, Torrance, CA 90501-1430, USA) column with a column guard, a 20 µL loop and a UV detector at 260 nm. The flow was set to 1 mL/min. The eluent was phosphate buffer 0.03M (pH 7.4) + tetrabutylammonium hydroxide (TBAH) 10 mM. For 1 L phosphate buffer in water (mQ): 3.53 g (NH_4_)_2_HPO_4_ + 0.76 g (NH_4_)H_2_PO_4_ + 2.6 mL of 40% TBAH solution were mixed in water. The phosphate buffer was filtered through a 500 mL Nalgene™ Rapid-Flow™ Sterile Disposable Filter Unit with PES 0.2 µM membrane prior to use. To quantify unmodified nucleotides monophosphate 1a–4a (Figure A1), sodium salts of NMPs were purchased by Millipore-Sigma Italy. Calibration curves were obtained using scalar solutions of each one (Adenosine 5′-monophosphate disodium salt 1a. Guanosine 5′- monophosphate disodium salt 2a. Cytidine 5′-monophosphate disodium salt 3a; Uridine 5′- monophosphate disodium salt 4a) in the range of concentration 0.00026–0.14 µg/µL. Calibration curve of all mononucleotides 1a–4a showed a good response linearity with a coefficient of correlation (R^2^) of 0.999. Fixed samples were spiked with each nucleotide-monophosphate-disodium salt to definitely identify the unmodified 1a–4a nucleotide peaks in HPLC analysis. Fixed samples were diluted 1:10 in phosphate buffer and injected in HPLC in triplicate analyses for a run time of 60–70 min.

Each HPLC analysis was carried out loading about 600–700 ng of digested material.

### 4.4. RNA Isolation

#### 4.4.1. RNA Isolation from Fixed Specimens

On average 35 mg of formalin, Bouin and RCL2 fixed tissues were cut on a cold surface and transferred into a cleaned 2 mL vial. The homogenization was performed with 50 µl of Lysis solution obtained by the Maxwell^®^ RSC RNA FFPE kit (Cat. No. AS1440; Promega, Madison, WI, USA) using a homogenizer (Ultra-Turrax T25 basic. IKA^®^-WERKE; Staufen, Germany). The homogenization was carried out in wet ice for 20 s for four times at 20,000 rpm. Successively, 200 µL of Lysis solution and 15 µL of proteinase K were added to the homogenized samples and processed following the instructions of Maxwell^®^ RSC RNA FFPE kit (Cat. No. AS1440). Total RNA was automatically eluted in 50 µL of Nuclease-free water, split into aliquots and stored at −80 °C. RNA concentration and purity were measured by absorbance through NanoDrop^TM^ ND-1000 spectrophotometer (Thermo Fischer Scientific; Waltham, MA, USA).

#### 4.4.2. RNA Isolation from Fixed and Paraffin-Embedded Specimens

RNA was isolated from fixed and paraffin-embedded mouse liver samples and from routine clinical tissues. With regards of clinical tissues, two Bouin’s fixed and paraffin-embedded high-grade ovarian cancer samples and ten cancer FFPE samples of different origin were used. Those specimens were collected at the National Cancer Institute of Aviano and at the University Hospital of Trieste. Informed consent was obtained from participants included in the study and ethical approval was obtained by the Institutional Review Board of CRO-Aviano (protocol number 1213, 24 January 2017) and by the ethical committee of the University of Trieste (report n. 17, 4 August 2008).

RNA was isolated from four 10 µm-thick sections of fixed and paraffin-embedded specimens using the Maxwell^®^ RSC instrument (Promega; Madison, WI, USA) following the instruction of the Maxwell^®^ RSC RNA FFPE kit (Cat. No. AS1440). The protocol of isolation includes a digestion step at 56 °C and a de-modification step of 1 h at 80 °C. After RNA isolation, samples were split into aliquots and stored at −80 °C. RNA quantification and purity detection were assessed by NanoDrop^TM^ ND-1000 spectrophotometer (Thermo Fischer Scientific; Waltham, MA, USA).

#### 4.4.3. RNA Isolation from Peripheral Blood

RNA was isolated from peripheral blood from healthy volunteers by the use of Tri-reagent (Cat. No T9424, Millipore-Sigma, Milano, Italy). Samples were split into aliquots and stored at −80 °C. RNA quantification and purity detection were assessed by NanoDrop^TM^ ND-1000 spectrophotometer (Thermo Fischer Scientific, Waltham, MA, USA). Afterwards, an aliquot of RNA was submitted to DNAse digestion.

### 4.5. DNase Digestion

Thirty µL of total RNA were treated with 20 U of DNase I (Cat. No. 04716728001; Roche. Mannheim, Germany) and 20 U of RNase Inhibitor (Cat. No. N8080119; Thermo Fisher Scientific. Waltham, MA, USA) at 25 °C for 20 min. Then the reaction was stopped with 4 µL of EDTA 25 mM at 65 °C for 10 min as already described [34]. Samples were split into aliquots and stored at −80 °C.

### 4.6. RNA Integrity

RNA integrity was assessed through automated electrophoresis by the Agilent 2100 Bioanalyzer instrument (Agilent Technologies; Santa Clara, CA, USA). One microliter of each sample was loaded into the sample well of the Agilent RNA 6000 Nano Chip (Cat. No. 5067-1529; Agilent Technologies; Santa Clara, CA, USA) which was previously filled with the gel-dye mixture and the 5 µL of RNA 6000 Nano Marker, as suggested by the manufacture’s instruction (Cat. No. 5067-1512; Agilent Technologies; Santa Clara, CA, USA). In addition, the distribution of different length fragments was measured by the relative abundance in comparison to the total RNA area. The five ranges were defined as follows: from 1 to 59 nucleotides (1), from 60 to 149 (2), from 150 to 299 (3), from 300 to 449 (4) and equal or over 450 (5).

### 4.7. Reverse Transcription and Real-Time PCR Assay

For mt-CO1gene amplification 200 ng of mouse RNA were reverse transcribed using 250 U M-MLV reverse transcriptase (Thermo Fischer Scientific, Waltham, MA, USA; Cat. No. 28025013), 1 mM dNTPs, 0.01 M DTT, 4.5 mM MgCl_2_ and 8 U RNase Inhibitors (Thermo Fischer Scientific, Waltham, MA, USA; Cat. No. E00382) as already reported [35]. The amplification reactions for the mouse mitochondrially encoded cytochrome c oxidase I (mt-CO1) gene (mt-CO1) consist of three individual qPCR analyses amplifying fragments of 60 (S-Small fragment), 179 (M-Medium fragment) and 302 bases (L-Long fragment) using already published primer sequences [18]. For each real-time PCR reaction, 20 ng of cDNA was added to 10 µL of Fast EvaGreen qPCR Master Mix 2× (Biotium, Fremont, CA, USA; Cat. No. 31003), 300 nM of reverse and forward primers (IDT, Coraville, IA, USA) in a final volume of 20 µL. Each reaction was run in duplicate on a Mastercycler^®^ ep Realplex (Eppendorf, Hamburg, Germany) using the following cycling conditions: 95 °C for 2 min, 45 cycles of 95 °C for 20 s, 30 s of annealing temperature and the extension at 72 °C for 30 or 45 s (extension time of 45 s was used for 302 bases amplicon). Annealing temperature was 60 °C for 60 bases amplicon, 57.5 °C for 179 bases amplicon and 61 °C for 302 bases amplicon. At the end of the protocol, the melting curve analysis was carried out to evaluate the specificity of the amplified products.

### 4.8. ∆Amp Analysis

The ∆Amp method [15] was applied to measure the difference in threshold cycles between amplicons of the mt-CO1 gene. According to the published method differences were calculated between threshold cycles related to the amplification of mt-CO1 M (179 bases) and S (60 bases) and between L amplicon (302 bases) S (60 bases). The method is based on mt-CO1 gene amplification, which is virtually resistant to RNases [15], therefore it can be used to monitor the chemical and physical degradation of RNAs.

### 4.9. Statistical Analysis

Linear regression was performed by plotting the Ct values versus the amplicon size for each pre-analytical condition. ANCOVA test was run to investigate on regression lines, in detail to test if slopes and intercepts were significantly different. Bonferroni analysis was carried out to inspect multiple comparison among all samples. All the statistical analyses were carried out using the GraphPad Prism 8.0 software (San Diego, CA, USA).

## Figures and Tables

**Table 1:** Results of the calibration curves: recovery percentage as ratio of the recovered amount from HPLC vs. the loaded one, results of the regression analysis, LRE (Linear Regression Equation) LOD (limit of detection) and LOQ (limit of quantification).

Variables	CMP	UMP	GMP	AMP
130 ng/µL	98 ± 1 ^1^	99 ± 1	98 ± 1	97 ± 1
65 ng/µL	98 ± 1	98 ± 1	98 ± 1	99 ± 1
39 ng/µL	98 ± 1	99 ± 1	95 ± 1	98 ± 1
13 ng/µL	97 ± 1	97 ± 1	93 ± 1	93 ± 1
1.3 ng/µL	91 ± 4	98 ± 2	90 ± 3	92 ± 3
LRE	5.8 × 10^−5^ × −3.9 × 10^−4^	4.6 × 10^−5^ × +8.4 × 10^−5^	4.6 × 10^−5^ × −1.0 × 10^−3^	2.5 × 10^−5^ × −2.8 × 10^−4^
R^2^	>0.999	>0.999	>0.999	>0.999
LOQ (ng/µL) 10 (σ/s) ^1^	7.1	4.4	14.9	11.2
LOD (ng/µL) 3.3 (σ/s)	2.3	1.4	4.9	3.8

^1^ standard deviation/noise.

**Table 2:** Retention times (RT) for each NMP and hm-NMP detected in the analytical sessions; n represents the number of detections, RT represents the retention time in minutes, in brackets are reported the coefficients of variation (CV).

Analytical Session	CMP		Hm-CMP		UMP		AMP		Hm-AMP		GMP		Hm-GMP	
	*n*	RT	*n*	RT	*n*	RT	*n*	RT	*n*	RT	*n*	RT	*n*	RT
1°—02/2019	*45*	6.0 (2.3)	*3*	7.8 (0.4)	*42*	8.2 (2.9)	*45*	28.2 (7.3)	*3*	46.3 (0.4)	*45*	12.0 (4.1)	*24*	22.1 (6.7)
2°—04/2019	*18*	5.8 (0.8)	*0*	-	*18*	7.9 (1.5)	*18*	26.1 (4.1)	*0*	-	*17*	11.5 (2.1)	*12*	20.8 (3.4)
3°—07/2019	*24*	5.8 (2.4)	*0*	-	*24*	7.8 (3.8)	*24*	25.9 (7.1)	*0*	-	*24*	11.3 (5.6)	*6*	21 (3.3)
4°—02/2020	*27*	5.4 (4.0)	*6*	6.5 (1.4)	*27*	7.1 (2.6)	*24*	21.9 (5.8)	*3*	40.8 (0.3)	*27*	10.1 (3.6)	*18*	18.5 (7.0)
5°—06/2020	*17*	5.3 (1.3)	*0*	-	*17*	6.8 (2.5)	*19*	20.35 (7.1)	*6*	34.1 (0.3)	*19*	9.52 (4.8)	*12*	17.5 (5.7)
6°—08/2020	*4*	5.2 (1.4)	*2*	6.2 (0.9)	*4*	6.6 (0.8)	*4*	19.0 (0.6)	*2*	28.3 (0.8)	*4*	9.04 (0.9)	*4*	16.05 (1.2)
Total	*135*	5.7 (5.4)	*11*	6.7 (9.4)	*132*	7.6 (7.7)	*134*	25.0 (14.1)	*14*	36.7 (17.6)	*134*	11.0 (9.8)	*79*	20.2 (12.2)

**Table 3:** Results of the digestion by S1 Nuclease of each polyNMP: recovery percentage as ratio of the recovered amount from HPLC vs. the loaded one and ratio between NMP and cNMP (cyclic NMP) areas.

Variables	CMP	UMP	GMP	AMP
PolyC	93 ± 1			
PolyU		91 ± 1		
PolyG			89 ± 1	
PolyA				92 ± 1
NMP/cNMP	8.6 ± 1	9.0 ± 1	10.7 ± 1	7.3 ± 1

**Table 4:** Amount in percentage area of unmodified nucleotide monophosphates identified in the first set of samples as detected by chromatograms.

Sample ^1^	CMP	UMP	GMP	AMP	TOT
Frozen	21 ± 1%	18 ± 1%	35 ± 1%	26 ± 1%	100 ± 1%
FF	21 ± 3%	17 ± 2%	26 ± 1%	21 ± 3%	85 ± 3%
RF	21 ± 1%	17 ± 1%	38 ± 1%	24 ± 1%	99 ± 1%
BF	21 ± 1%	18 ± 1%	37 ± 1%	24 ± 1%	100 ± 1%
FFPE	15 ± 1%	16 ± 1%	28 ± 1%	22 ± 1%	80 ± 1%
RFPE	21 ± 3%	15 ± 1%	32 ± 3%	20 ± 1%	88 ± 5%
BFPE	21 ± 1%	16 ± 1%	38 ± 1%	21 ± 2%	96 ± 1%

^1^ FF Formalin-fixed, RF RCL-2 fixed, BF Bouin’s fixed, FFPE Formalin-fixed and paraffin-embedded, RFPE RCL-2 fixed and paraffin-embedded, BFPE Bouin’s fixed and paraffin-embedded.

**Table 5:** Results of RNA quantification and integrity detection by BioAnalyzer in mouse livers.

Samples ^1^	A260/A280	A260/A230	RIN
Frozen	2.10	2.07	4.0
FF liver	1.95	2.14	2.1
BF liver	1.70	2.09	N.A. ^2^
RF liver	2.09	2.12	2.2
FFPE liver	2.02	2.12	2.2
BFPE liver	1.93	2.14	2.4
RFPE liver	2.05	2.11	1.7

^1^ FF Formalin-fixed, BF Bouin’s fixed, RF RCL-2 fixed, FFPE Formalin-fixed and paraffin-embedded, BFPE Bouin’s fixed and paraffin-embedded, RFPE RCL-2 fixed and paraffin-embedded. ^2^ N./A. not assessable.

**Table 6:** Results of the regression analysis for mt-CO1 amplification by RT-qPCR.

	Frozen	FF	BF	RF	FFPE	BFPE	RFPE	*p* ^1^
Slope	0.01	0.06	0.05	0.04	0.03	0.09	0.03	0.14
Y-intercept	15.08	15.50	28.14	13.20	14.04	17.50	14.40	<0.0001
R^2^	0.97	0.96		0.86	0.92		0.9	

^1^ Results of the ANCOVA test. R^2^ values were not reported for BF and BFPE as the lines were generated by two values.

**Table 7:** Results of HPLC analysis as area percentage of canonical NMPs and integrity of clinical samples. Samples’ age is reported in years.

Sample	Sample	Age	RIN	CMP	UMP	GMP	AMP	TOT
1—HGSOC B1	BFPE	11	1.7	19%	18%	35%	24%	96%
2—HGSOC B2	BFPE	10	2.4	19%	17%	34%	24%	94%
3—HGSOC	FFPE	9	2.3	12%	15%	23%	18%	63%
4—Breast 1	FFPE	30	2.4	16%	17%	31%	24%	88%
5—Breast 2	FFPE	28	2.4	22%	17%	33%	25%	97%
6—Colon 1	FFPE	20	N.A. ^1^	19%	16%	32%	22%	89%
7—Colon 2	FFPE	18	2.0	22%	17%	33%	23%	95%
8—Glioma	FFPE	11	2.2	18%	15%	34%	25%	92%
9—Melanoma	FFPE	13	2.5	20%	16%	35%	24%	95%
10—Pancreas	FFPE	15	2.4	20%	14%	36%	19%	89%
11—Prostate	FFPE	18	2.3	19%	17%	35%	26%	97%
12—Uterine cervix	FFPE	28	2.2	6%	11%	10%	4%	31%
13—Blood 1	Na_2_EDTA	0	9.1	20%	17%	33%	30%	100%
14—Blood 2	Na_2_EDTA	0	8.8	20%	17%	33%	30%	100%
15–Blood 3	Na_2_EDTA	0	8.6	20%	18%	33%	29%	100%
Standard RNA	Solution	-	9.7	22%	17%	34%	27%	100%

^1^ N.A. not assessable.

**Figure A3:**
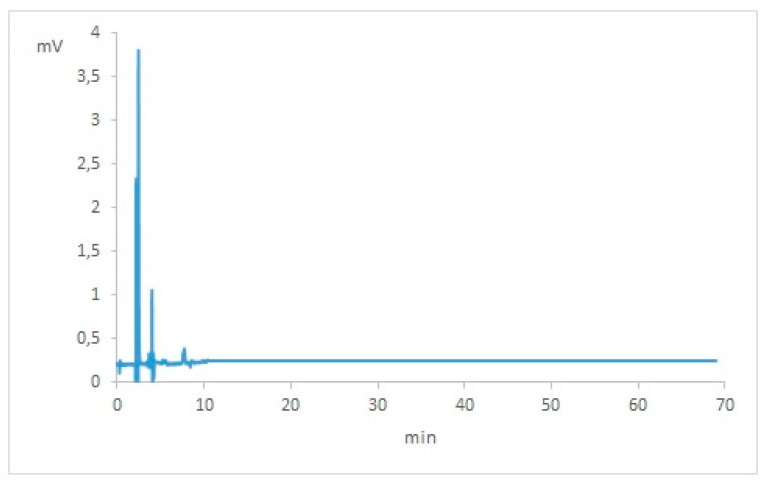
Chromatogram of no template control of the digestion reaction including S1 nuclease and its 1x reaction buffer.

## Supplementary Materials

Supplementary materials can be found at https://www.mdpi.com/1422-0067/21/20/7540/s1. Figure S1 Gel-like images of the BioAnalayzer runs of (**a**) 1—ladder and mouse livers submitted to different treatment: 2—Fresh frozen; 3—24 h in neutral buffered formalin (FF); 4—24 h in neutral buffered formalin and paraffin embedded (FFPE); 5—24 h in Bouin’s solution (BF); 6—24 h in Bouin’s solution and paraffin embedded (BFPE); 7—24 h in RCL2 (RF); 8—24 h in RCL2 and paraffin embedded (RFPE); (**b**) 1—ladder; and clinical samples 2—HGSOC B1; 3—HGSOC B2; 4—HGSOC; 5—Breast 1 and 6—Breast 2; (**c**) 1—ladder; and clinical samples 2—Colon 1; 3—Colon 2; 4—Blood 1; 5—Blood 2; (**d**) 1—ladder; and clinical samples 2—Glioma; 3—Melanoma; 4—Pancreas; 5—Prostate; 6—Uteral Cervix and 7—Blood 3. RIN values are reported in Table 5 and Table 7 of the main document.

## Abbreviations

BF	Bouin’s solution fixed
BFPE	Bouin’s solution fixed and paraffin-embedded
c-NMP	Cyclic-NMP
Ct	Threshold cycle
FF	Formalin-fixed
FFPE	Formalin-fixed and paraffin-embedded
Hm-NMP	Hydroxymethyl- nucleotide monophosphate
HPLC	High performance liquid chromatography
NMP	Nucleotide monophosphate
Mt-CO1	Mitochondrially encoded cytochrome C oxidase I
RIN	RNA integrity number
RF	RCL2^®^ fixed
RFPE	RCL2^®^ fixed and paraffin-embedded
Rt-PCR	Reverse transcription-polymerase chain reaction

## Appendix A

The main step in formalin fixation process is the formation of hydroxymethyl adducts to nitrogenous bases. Hydroxymethyl-AMP, -CMP, -UMP and –GMP are not commercially available, therefore they were synthesized in vitro. The main target was to verify that those species do not overlap with unmodified nucleotides monophosphate in HPLC analysis. Consequently, the synthesis and characterization of those hydroxymethyl adducts is reported here below.

### Appendix A.1. Synthesis of Hydroxymethyl-AMP, -CMP, -UMP and –GMP

Synthesis was carried out as already described [26]. Briefly, 10 mL of 20 mM solution of each nucleotide-monophosphate- disodium salt in 4% neutral buffered formaldehyde was generated and left for 1 week at room temperature in dark. The crude mixtures referring to the 4 reactions were precipitated from ice cold 2% LiClO_4_ in acetone and centrifuged for 15 min at 1000× *g* at 4 °C. The supernatant liquid was discarded and the precipitates were washed twice with ice-cold acetone. The crude mixture was evaporated to dryness under vacuum and stored at −20 °C up to HPLC and ^1^H-NMR analysis.

### Appendix A.2. 1H-NMR Characterization of Hydroxymethyl-AMP, -CMP, -UMP and –GMP

^1^H-NMR spectra were recorded on a Varian 400-MR spectrometer using D_2_O as solvent (calibration was set on H_2_O signal at 4.65 ppm). Coupling constants (J) are given in Hz. Analysis of the NMR spectra was performed using the MestReNova NMR data processing of Mestrelab Research S.L. ^1^H-NMR spectra are related to the structures reported in Figure A1.

**AMP 1a** (sodium salt) ^1^H NMR (400 MHz, D_2_O), (ppm): 3.97 (m, H-5′), 4.24 (m, H-4′), 4.36 (dd, J_1_ 3.7, J_2_ 5.1, H-3′), 5.98 (d, J 5.8, H-1′), 8.08 (s, H-8), 8.34 (s, H-2). Chemical shifts are in accordance with the literature [9].

**Hm-AMP 1b** in admixture with **1a** (66% of **1b**) ^1^H NMR (400 MHz, D_2_O), δ (ppm): 3.86 (m, H-5′, **1a** + **1b**), 4.21 (m, H-4′, **1a** + **1b**), 4.36 (m, H-3′, **1a** + **1b**), 5.01 (s, NHCH_2_OH, **1b**), 5.98 (d, J 5.8, H-1′, **1a**), 6.00 (d, J 5.9, H-1′, **1b**), 8.09 (s, H-8, **1a**), 8.19 (s, H-8, **1b**), 8.45 (s, H-2, **1a**), 8.48 (s, H-2, **1b**).

**GMP 2a** (sodium salt) ^1^H NMR (400 MHz, D_2_O), (ppm): 3.85 (m, H-5′), 4.18 (m, H-4′), 4.33 (m, H-3′), 5.76 (d, J 5.8, H-1′), 8.05 (s, H-8).

**Hm-GMP 2b** in admixture with **2a** (36% of **2b**) ^1^H NMR (400 MHz, D_2_O), (ppm): 3.86 (m, H-5′, **2a** + **2b**), 4.21 (m, H-4′, **2a** + **2b**), 4.36 (m, H-3′, **2a** + **2b**), 4.79 (apparent d, J 4.4, NHCH_2_OH, **2b**), 5.75 (d, J 6.0, H-1′, **2a**), 5.88 (d, J 5.9, H-1′, **2b**), 8.03 (s, H-8, **2a**), 8.04 (s, H-8, **2b**).

**CMP 3a** (sodium salt) ^1^H NMR (400 MHz, D_2_O), (ppm): 3.83 (ddd, J_1_ 11.9, J_2_ 5.0, J_3_ 2.9, H-5′), 3.91 (ddd, J_1_ 11.9, J_2_ 3.7, J_3_ 2.9, H-5′), 4.10 (m, H-4′), 4.21 (m, H-3′ + H-2′), 5.87 (d, J 3.9, H-1′), 6.00 (d, J 7.6, H-5), 7.98 (d, J 7.6, H-6).

**Hm-CMP 3b** in admixture with **3a** (50% of **3b**) ^1^H NMR (400 MHz, D_2_O), (ppm): 3.83 (m, H-5′, **3a** + **3b**), 3.91 (m, H-5′, **3a** + **3b**), 4.09 (m, H-4′, **3a** + **3b**), 4.18 (m, H-3′ + H-2′, **3a** + **3b**), 5.84 (m, H-1′, **3a** + **3b**), 4.74 (s, NHC*H*_2_OH, **3b**), 5.96 (d, J 7.6, H-5, **3b**), 5.97 (d, J 7.6, H-5, **3a**), 7.92 (d, J 7.6, H-6, **3a**), 7.94 (d, J 7.6, H-6, **3b**).

**UMP 4a** (sodium salt) ^1^H NMR (400 MHz, D_2_O), (ppm): 3.82 (ddd, J_1_ 11.8, J_2_ 5.0, J_3_ 3.0, H-5′), 3.88 (ddd, J_1_ 11.8, J_2_ 3.7, J_3_ 3.0, H-5′), 4.11 (m, H-4′), 4.21 (t, J 4.2, H-3′), 4.28 (t, J 5.2, H-2′), 5.84 (d, J 8.1, H-5), 5.85 (d, J 5.2, H-1′), 7.98 (d, J 8.1, H-6).

As observed by their ^1^H-NMR spectra, **1b**–**3b** showed a characteristic peak at 5.01 ppm, 4.79 ppm and 4.74 ppm respectively, corresponding to the CH_2_ protons of the NHCH_2_OH group obtained after reaction with formaldehyde. The formylation reaction was in any event, not complete and a mixture of both unmodified **1a**–**3a** and their corresponding hydroxylmethyl derivatives **1b**–**3b** was obtained. Chemical shifts of all protons were different in all cases although overlapping was observed in some cases. To estimate the percentage of formylation for AMP the area of the H-8 signal of **1a** (8.45 ppm) and **1b** (8.48 ppm) was correlated giving 66% in favor of 1b. For GMP we referred to H-1′ signal since they were well separated in the two compounds (5.75 ppm for **2a** and 5.88 ppm for **2b**) giving a conversion of 36% for compound **2b**. For CMP we referred to the hydroxymethyl signal of **3b** and the H-1′ signal which is the sum of both **3a** and **3b** giving a conversion of 50%. In the case of UMP no signal appeared in the range 4.70–5.50 ppm indicating that no formylation occurred. To confirm, all the other signals were not split as no hydroxymethyl derivative was formed. This is in accordance with the structure of UMP which hasn’t got any amino group, while **1a**–**3a** have an amino group in the heterocyclic ring which can react with formaldehyde.

### Appendix A.3. HPLC Characterization of Hydroxymethyl-AMP, -CMP, -UMP and –GMP

The crude mixtures of hydroxymethyl-nucleotide were dissolved in phosphate buffer and injected in HPLC as triplicate analysis (Figure A2).

## Appendix B

∆Amp was calculated between amplicons of mt-CO1. ∆Amp M-S refers to the difference between Ct detected for 179 b amplicon and 60 b amplicon of mt-CO1, ∆Amp L-S refers to the difference between Ct detected for 302 b amplicon and 60 b amplicon of mt-CO1, as already defined [15].

**Table A1:** ∆Amp of mt-CO1 gene for mouse livers samples.

Specimen	∆Amp M-S (60–179)	∆Amp L-S (302–60)
Frozen	1.00	2.93
FF	4.43	13.48
BF	5.63	N.A. ^1^
RF	1.48	10.05
FFPE	1.50	6.37
BFPE	10.41	N.A.
RFPE	1.44	6.87

^1^ N.A. not assessable

## Appendix C

In order to investigate on the effect of de-modification step of RNAs, RNA isolation by the use of the RNA isolation procedure for FFPE (See Material and method section) was carried out parallelly using 2 slides of 10 µm thick slides of FFPE prostate cancer tissues. One replicate included the RNA de-modification step at 80 °C for 1 h, while the in the other the step was omitted. Although hm-AMP and hm-CMP were not detectable a reduction of the hm-GMP (up to ten times) was obtained after the de-modification (Figure A4). This step did not influence the BioAnalyzer analysis, which returned a RIN value of 2.3 for both (Figure A5).
