# Supplementary material for: A Novel HPLC-Based Method to Investigate on RNA after Fixation
Source: Int J Mol Sci. 2020 Oct 13;21(20):7540. doi: 10.3390/ijms21207540 (PMC7588918; doi:10.3390/ijms21207540)
Supplement: Supplementary file 1 [file ijms-21-07540-s001.pdf]

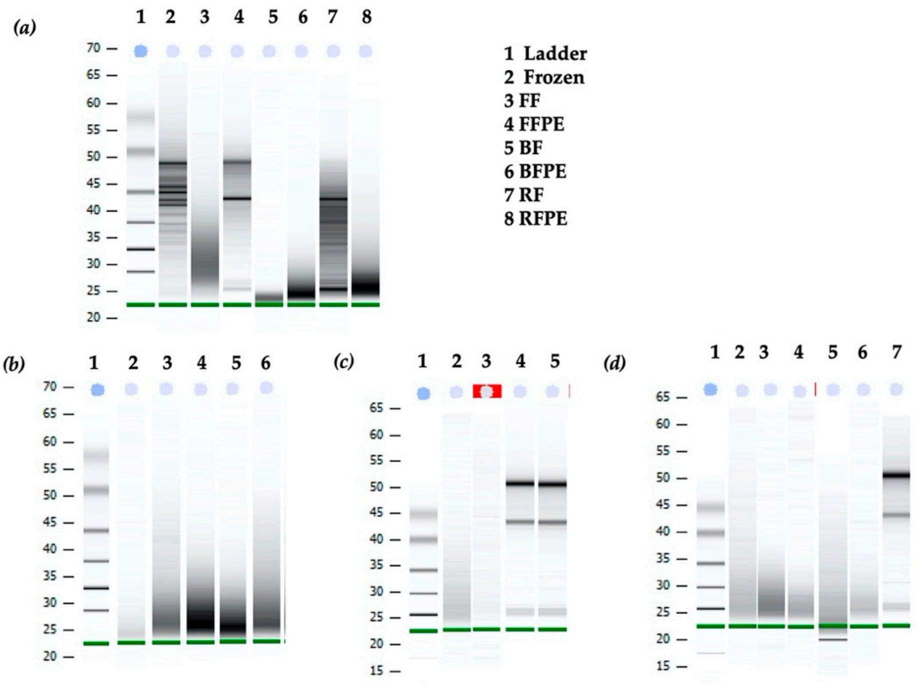

Supplementary Figure S1. Gel-like images of the BioAnalayzer runs of ***(a)*** 1- ladder and mouse livers submitted to different treatment: 2- Fresh frozen; 3- 24 hours in neutral buffered formalin (FF); 4- 24 hours in neutral buffered formalin and paraffin embedded (FFPE); 5- 24 hours in Bouin's solution (BF); 6- 24 hours in Bouin's solution and paraffin embedded (BFPE); 7- 24 hours in RCL2 (RF); 8- 24 hours in RCL2 and paraffin embedded (RFPE); ***(b)*** 1- ladder ; and clinical samples 2-HGSOC B1; 3- HGSOC B2; 4- HGSOC; 5- Breast 1 and 6- Breast 2; ***(c)*** 1- ladder ; and clinical samples 2- Colon 1; 3- Colon 2; 4- Blood 1; 5- Blood 2; ***(d)*** 1- ladder ; and clinical samples 2-Glioma; 3- Melanoma; 4- Pancreas; 5- Prostate; 6- Uteral Cervix and 7- Blood 3. RIN values are reported in Table 5 and Table 7 of the main document.
